# Higher *FOXP3*-TSDR demethylation rates in adjacent normal tissues in patients with colon cancer were associated with worse survival

**DOI:** 10.1186/1476-4598-13-153

**Published:** 2014-06-18

**Authors:** Changhua Zhuo, Zhiyuan Li, Ye Xu, Yuwei Wang, Qingguo Li, Junjie Peng, Hongtu Zheng, Peng Wu, Bin Li, Sanjun Cai

**Affiliations:** 1 Department of Colorectal Surgery, Fudan University Shanghai Cancer Center; Department of Oncology, Shanghai Medical College, Fudan University, No. 270 Dong-an Road, Shanghai 20032, People’s Republic of China; 2 Key Laboratory of Molecular Virology & Immunology, Unit of Molecular Immunology, Institut Pasteur of Shanghai, Shanghai Institutes for Biological Sciences, Chinese Academy of Sciences, No. 320 Yue-yang Road, Shanghai 20031, People’s Republic of China; 3 Department of Surgical Oncology, Fujian Provincial Cancer Hospital, Teaching Hospital of Fujian Medical University, No. 420 Fu-ma Road, Fuzhou 350014, People’s Republic of China

**Keywords:** Regulatory T cells, Colorectal cancer, Transcription factor forkhead box P3, Treg-specific demethylated region, Survival analysis, Cox regression

## Abstract

**Background:**

The influence of natural regulatory T cells (nTregs) on the patients with colon cancer is unclear. Demethylated status of the Treg-specific demethylated region (TSDR) of the *FOXP3* gene was reported to be a potential biomarker for the identification of nTregs.

**Methods:**

The demethylation rate of the TSDR (TSDR-DMR) was calculated by using methylation-specific quantitative polymerase chain reaction (MS-qPCR) assay. The expression of TSDR-DMR and *FOXP3* mRNA was investigated in various colorectal cancer cell lines. A total of 130 colon carcinoma samples were utilized to study the DMR at tumor sites (DMR_T_) and adjacent normal tissue (DMR_N_). The correlations between DMRs and clinicopathological variables of patients with colon cancer were studied.

**Results:**

The TSDR-DMRs varied dramatically among nTregs (97.920 ± 0.466%) and iTregs (3.917 ± 0.750%). Significantly, DMR_T_ (3.296 ± 0.213%) was higher than DMR_N_ (1.605 ± 0.146%) (n = 130, *p* = 0.000). Higher DMR_N_ levels were found in female patients (*p* = 0.001) and those with distant metastases (*p* = 0.017), and were also associated with worse recurrence-free survival in non-stage IV patients (low vs. high, *p* = 0.022). However, further Cox multivariate analysis revealed that the *FOXP3*-TSDR status does not have prognostic value.

**Conclusion:**

MS-qPCR assays of *FOXP3*-TSDR can efficiently distinguish nTregs from non-nTregs. Abnormal recruitment of nTregs occurs in the local tumor microenvironment. Infiltration of tissue-resident nTregs may have a negative role in anti-tumor effects in patients with colon cancer; however, this role is limited and complicated.

## Background

Colorectal cancer (CRC) is one of the most prevalent life-threatening malignancies, ranking as the third most frequently diagnosed cancer and the second leading cause of cancer death in the United States [1]. Although survival depends mainly on the stage at diagnosis [2,3], one of the increasingly explored therapeutic options for CRC is the modulation of the immune system [4].

Regulatory T cells (Tregs) are essential for maintaining self-tolerance [5,6]. The transcription factor forkhead box P3 (FOXP3) is regarded as a critical developmental and functional factor for CD4 ^+^ CD25^+^ Tregs [5,7,8]. In humans, however, FOXP3 is not expressed exclusively in natural Tregs (nTregs) [9-13]. Recently, it was shown that the Treg-specific demethylated region (TSDR) is significantly demethylated in human nTregs, while it is completely methylated in induced Tregs (iTregs) and other non-suppressive T cells that also express FOXP3 [14,15]. TSDR is a CpG dinucleotide-rich and highly conserved region within the conserved non-coding sequences 2 (CNS2), located in the first intron of the *FOXP3* gene. Demethylation in TSDR is thought to contribute to both the stability of FOXP3 expression and the maintenance of the suppressive phenotype for nTregs [16]. FOXP3 induction by TGF-β is associated with only partial or no demethylation of the TSDR, an unstable state that is reversed upon restimulation [15,17].

At present, the exact quantification methods of human Tregs, which are based on the expression of the FOXP3 protein, are technically demanding and error-prone, and interpretation of the results can be ambiguous and subjective [4,10]. These methods include tissue microarrays (TMA), immunohistochemistry (IHC), and flow cytometry (FCM). Moreover, it is impossible to differentiate nTregs from non-Treg cells using these traditional methods. A methylation-specific quantitative polymerase chain reaction (MS-qPCR) assay was recently developed to quantify the proportion of demethylated *FOXP3*-TSDR in the peripheral blood as well as in solid tissue samples in various diseases, and it was regarded as a potential biomarker for the identification of nTregs [4,14,15,17-21].

Tregs are considered to be a major cell population involved in tumor immune tolerance [22]. Elevated proportions of Tregs infiltrating the tumor nests or in the peripheral blood have been described in several neoplasms; and generally, this appeared to be associated with unfavorable clinical outcomes [23-26]. In patients with CRC, however, there have been several discordant results on the prognostic value of Treg infiltration, which might play a negative [27-30] or positive [31-34] role in combating cancer. The influence of nTregs, the most important subset of Tregs, on the survival outcomes of patients with CRC remains unclear.

In this study, we aimed to investigate the correlation between the demethylation status of *FOXP3*-TSDR and the clinicopathological features of Chinese patients with colon cancer. We determined that the established MS-qPCR system could function well in identification of nTregs from non-nTregs. Our data indicated that nTregs might have a negative role in anti-tumor effects, although their impacts on the survival outcomes of patients with colon cancer may be limited and complicated.

## Results

### The MS-qPCR system could efficiently differentiate nTregs from non-nTregs using the *FOXP3*-TSDR demethylation assay

**Figure 1:**
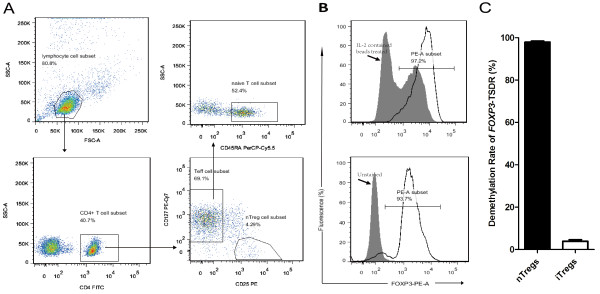
**Purification of nTregs and iTregs from PBMCs and their identification using *FOXP3*-TSDR demethylation-specific qPCR assays. (A)** Purification of CD4^+^CD25^+^CD127^lo^ nTregs (bottom right) and CD4^+^CD25^-^CD45RA^+^ naïve T cells (upper right) by FACS after separation from PBMCs obtained from a male donor. **(B)** Representative analysis of the cell purities of FOXP3^+^ cells in nTregs (93.7%, lower panel) and iTregs (97.2%, upper panel) purified from a CD4^+^ CD25^+^ T cell subset by FACS after Treg expansion *in vitro*. As indicated with black arrows, populations of unstained cells and cells containing IL-2 beads were used as the controls for the quantification of nTregs and iTregs, respectively. **(C)** The demethylation rates of *FOXP3*-TSDR varied dramatically between nTregs and iTregs, and the specific primer sets were verified in quantitative real-time PCR.

Human CD4^+^CD25^+^CD127^lo^ nTregs and CD4^+^CD25^-^CD45RA^+^ naïve T cells from a male donor were purified after separation from peripheral blood mononuclear cells (PBMCs) by fluorescence-activated cell sorting (FACS), and iTregs were differentiated from the population of naïve T cells (Figure 1A). Both the nTregs and iTregs were expanded *in vitro*, achieving a cell purity of more than 93% (Figure 1B).To verify whether the primer sets functioned properly, we quantitatively analyzed the demethylation rates (DMR) of TSDR in nTregs and iTregs. The amplification products were verified by DNA sequencing. The TSDR-DMR varied dramatically among nTregs (97.920 ± 0.466%) and iTregs (3.917 ± 0.750%) (Figure 1C). The ideal 100% and 0% TSDR-DMR could not be achieved for nTregs and non-nTregs, respectively, due to the cell purities mentioned above. Our data confirmed that the MS-qPCR assay coupled with the specific primer sets could efficiently distinguish FOXP3+ nTregs from non-nTregs.

### CRC cell Lines exhibit low levels of demethylated *FOXP3*-TSDR

**Figure 2:**
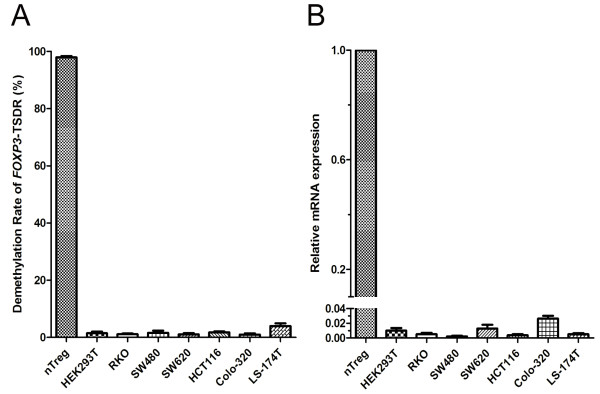
**CRC cell lines express very low levels of demethylated *FOXP3-*TSDR and *FOXP3 *mRNA.** HEK 293T cells, which do not express *FOXP3* mRNA, served as negative controls. **(A)** Compared to nTregs, CRC cell lines showed lower levels of demethylated *FOXP3*-TSDR based on an MS-qPCR assay. **(B)** Extremely low levels of *FOXP3* mRNA expression were observed among CRC cell lines when compared to nTregs based on a real-time RT-PCR assay.

We investigated the TSDR-DMR among various cell lines. Among HEK 293T cells, which do not express FOXP3 and thus served as negative controls here, an extremely low DMR (1.484 ± 0.579%) was documented. When compared to nTregs, an extremely low DMR was also detected among CRC cell lines, ranging from a mean of 0.974% (for Colo-320) to a mean of 4.003% (for LS-174T) (Figure 2A). Compared to its level in nTregs, extremely low levels of *FOXP3* mRNA expression (Figure 2B) and undetectable protein expression (data not shown) were consistently detected among HEK 293T and CRC cell lines. These results reinforced the notion that CRC cells barely express this biomarker and make a very limited contribution to the overall demethylation status in tumor tissues.

### Analysis of the *FOXP3*-TSDR demethylation status in solid tissue samples revealed abnormal recruitment and predominant enrichment of nTregs in the tumor microenvironment

Next, we evaluated the demethylation status of solid tissue samples obtained from patients with colon cancer. For a specific patient, compared to the corresponding adjacent normal tissues, tumor tissues contain the altered proportion of nTregs aside from the extra malignant cells. Because CRC cells scarcely express this epigenetic marker for nTregs (as described above), the evaluation of the general *FOXP3*-TSDR demethylation status in the tissue sample by MS-qPCR can reveal the density of nTregs within the parenchymal tissue in each sample.

**Figure 3:**
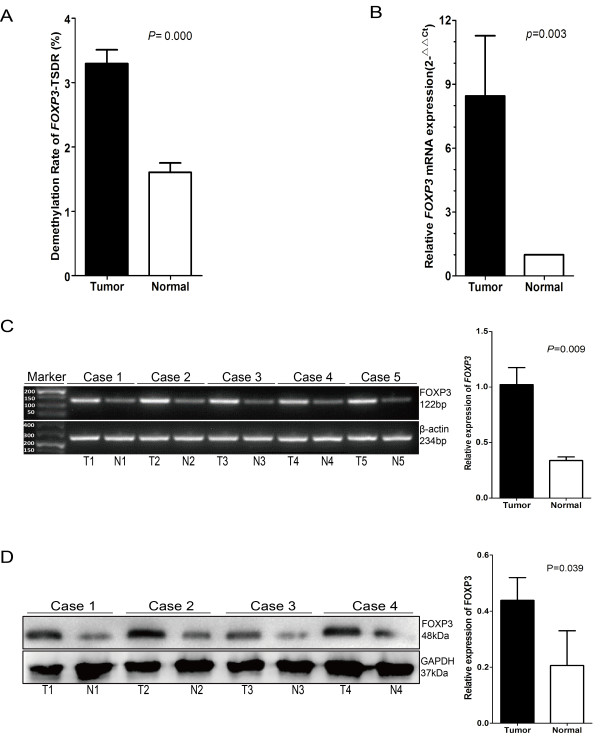
**TSDR-DMR, expression levels of *FOXP3 *mRNA and protein in tissue samples. (A)** Significantly higher TSDR-DMR in tumor samples versus normal samples, as investigated by MS-qPCR (*p* = 0.000, n = 130). (B) Significantly higher *FOXP3* mRNA expression in samples of tumor tissues versus samples of normal tissues, as detected by real-time RT-PCR (*p* = 0.003, n = 21). The Wilcoxon matched pairs test was applied to both **(A)** and **(B)** because the data were not normally distributed. A two-tailed *p* value ≤0.05 was considered statistically significant. **(C)** Representative results showing higher *FOXP3* mRNA expression in tumor tissues versus normal tissues, as detected by agarose gel electrophoresis (*p* = 0.009, n = 5). RT-PCR products of target (*FOXP3*) and internal control (*β-actin*) gene were loaded in two rows of parallel lanes and electrophoreses were performed simultaneously. DL500 was served as a DNA marker. **(D)** Representative result showing increased FOXP3 protein synthesis in tumor tissues versus normal tissues, as detected by Western blotting (*p* = 0.039, n = 4). For both **(C)** and **(D)**, ImageJ software was used to perform quantitative analysis of the densities of band, and paired *t* tests were applied. A two-tailed *p* value ≤0.05 was considered statistically significant.

Overall, a significantly higher TSDR-DMR was found in tumor sites versus normal sites (3.296 ± 0.213% vs. 1.605 ± 0.146%, n = 130, *p* = 0.000; Figure 3A). Furthermore, there existed significantly more *FOXP3* mRNA expression (8.454 vs. 1.000, n = 21, *p* = 0.003; Figure 3B) and higher protein synthesis in tumor tissues (see the representative differential expression of mRNA and protein shown by Figure 3C and Figure 3D). It implied that more Tregs accumulate in tumor nests than that in adjacent normal ones. Taken together, it is indicated that a local immune response, occurring mainly in tumor nests, is caused by the tissue-resident lymphocytes, including effector T cells and suppressive Tregs. Of the latter ones, nTregs were abnormally recruited and predominantly enriched within tumor microenvironment.

### The *FOXP3*-TSDR DMR was higher in normal tissues in female patients and those with distant metastases

Next, we investigated the associations between *FOXP3*-TSDR demethylation levels and the clinicopathological variables in the included patients. In addition to DMR_T_ and DMR_N_, the relative demethylation level in paired tumor tissue versus the corresponding adjacent normal colonic tissue (DMR_T_/DMR_N_) was also calculated for each patient.

**Figure 4:**
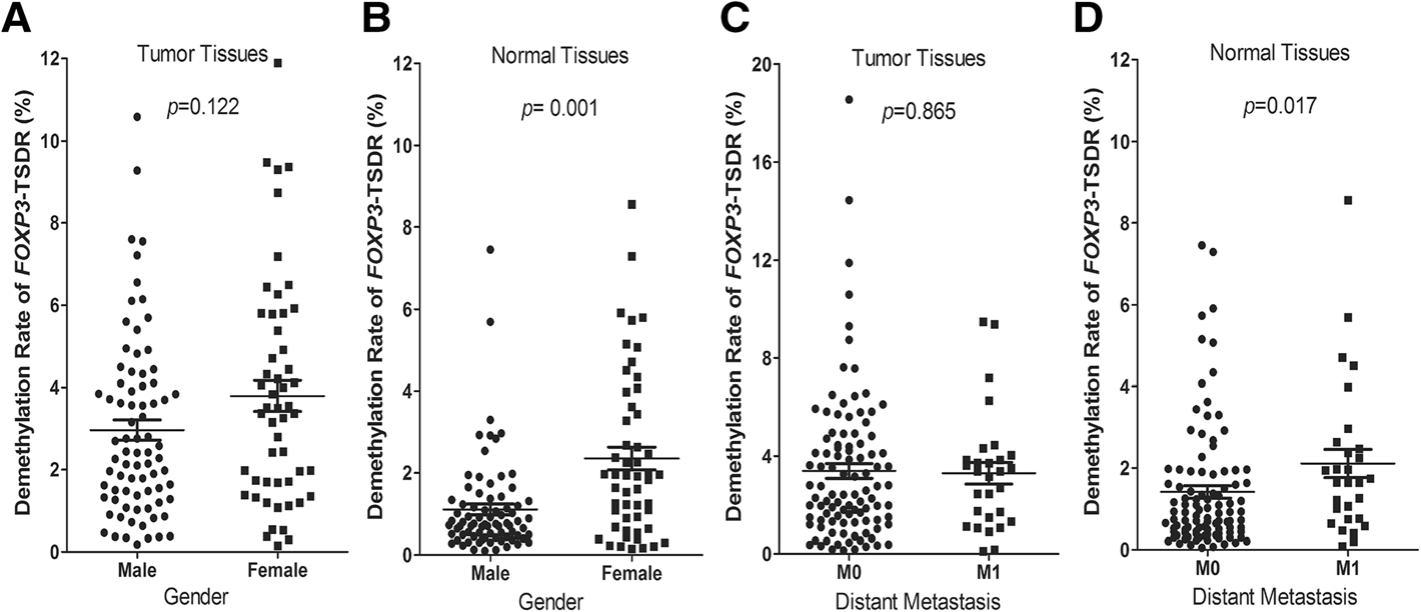
**Certain clinicopathological features were associated with the demethylation status of *FOXP3*-TSDR in tissue samples.** Associations between DMRs and Gender and Distant metastasis (M) were shown. A higher TSDR-DMR was detected in normal tissues (DMR_N_) both in female patients (*p* = 0.001, Figure 4**B**) and those with distant metastases (M1) (*p* = 0.017, Figure 4**D**). Either for Gender or for Distant metastasis, no correlation was found to be with the TSDR-DMR in tumor nests (DMR_T_) (Figure 4**A**, 4**C**). Mann-Whitney U tests were applied, and a two-tailed *p* value ≤0.05 was considered statistically significant.

Significantly, higher TSDR-DMR levels in normal tissues (DMR_N_) were found among female patients (*p* = 0.001) and those who had distant metastases (M1) (*p* = 0.017). Similarly, trends of higher DMR levels were also found in these two groups of patients according to DMR_T_, although the differences were not significant (*p* = 0.122 for Gender, and *p* = 0.865 for Distant metastases) (Figure 4). The relative demethylation levels (DMR_T_/DMR_N_), however, turned out to be significantly opposite to the results mentioned above (higher for male patients and those without distant metastases (M0), *p* = 0.003 *and* 0.037, respectively (Table 1). Other clinicopathological variables were also investigated; however, none were associated with the demethylation status of *FOXP3-*TSDR (Table 1). Our data here implied that nTregs in adjacent normal tissues might have a negative effect on the anti-tumor response.

**Table 1:** Relationships between the TSDR-DMRs and the clinicopathological features of patients with colon cancer

**Clinicopathological variables**		**N**	**DMR**_**T **_**(%)**		**DMR**_**N **_**(%)**		**DMR**_**T/**_**DMR**_**N**_	
			**Mean rank**	***p *** **value**	**Mean rank**	***p *** **value**	**Mean rank**	***p *** **value**
Gender	Male	78	61.33	0.122	56.72	0.001*	73.59	0.003*
	Female	52	71.76		78.66		53.37	
Age (years)	≤60	49	57.32	0.054	64.21	0.762	60.98	0.287
	>60	81	70.45		66.28		68.23	
Maximum Size (cm)	≤5	93	64.68	0.693	66.60	0.599	64.74	0.716
	>5	37	67.57		62.74		67.41	
Location^a^	Left side	62	59.76	0.097	62.07	0.322	64.48	0.769
	Right side	68	70.74		68.63		66.43	
Differentiation	G1-G2	87	68.45	0.204	66.78	0.581	65.34	0.947
	G3-G4	43	59.53		62.91		65.81	
Mucinous or signet-ring carcinoma	No	103	67.98	0.701	64.97	0.754	66.53	0.541
	Yes	27	64.85		67.52		61.56	
CEA	Normal	59	58.58	0.056	58.43	0.051	64.86	0.859
	Elevated	71	71.25		71.37		66.04	
Tumor (T) stage	T1-2	20	64.84	0.640	64.82	0.631	64.46	0.460
	T3-4	110	69.13		69.23		71.23	
Nodal (N) status	N0	63	66.97	0.216	65.76	0.275	68.72	0.595
	N1-2	66	62.32		64.93		58.50	
Distant metastases (M)^b^	M0	101	65.20	0.865	61.26	0.017*	69.18	0.037*
	M1	29	66.55		80.26		52.67	
AJCC stage	I-II	59	64.54	0.754	66.37	0.776	61.56	0.198
	III-IV	71	66.62		64.48		70.09	
Lymphovascular invasion	No	89	66.97	0.513	65.76	0.906	68.72	0.150
	Yes	41	62.32		64.93		58.50	
Perineural invasion	No	109	65.61	0.939	64.28	0.398	67.35	0.202
	Yes	21	64.93		71.86		55.90	
Extranodal tumor deposits	No	107	65.83	0.831	65.08	0.786	66.34	0.585
	Yes	23	63.98		67.43		61.61	

*A two-tailed *p* value ≤0.05 was considered statistically significant. Normality tests revealed that none of the data for these three variables (DMR_T_ (%), DMR_N_ (%), and DMR_T/_DMR_N_) were normally distributed; thus, the statistical analyses were performed using nonparametric Mann-Whitney U tests.

^a^The left side of the colon consists of the recto-sigmoid junction, sigmoid colon, descending colon, and splenic flexure. The right side of the colon consists of the cecum, ascending colon, hepatic flexure, and transverse colon.

^b^In this study, we included patients with resectable primary lesions. Patients who had distant metastases that were either resectable or unresectalbe were included.

*Abbreviations*: DMR_T_, demethylation rate in tumor tissues; DMR_N_, demethylation rate in normal tissues; CEA, carcinoembryonic antigen; AJCC, American Joint Committee on Cancer (AJCC).

### Clinical survival analysis revealed that non-stage IV patients with higher TSDR-DMR in normal Tissues (DMR_N_) had worse recurrence-free survival

**Figure 5:**
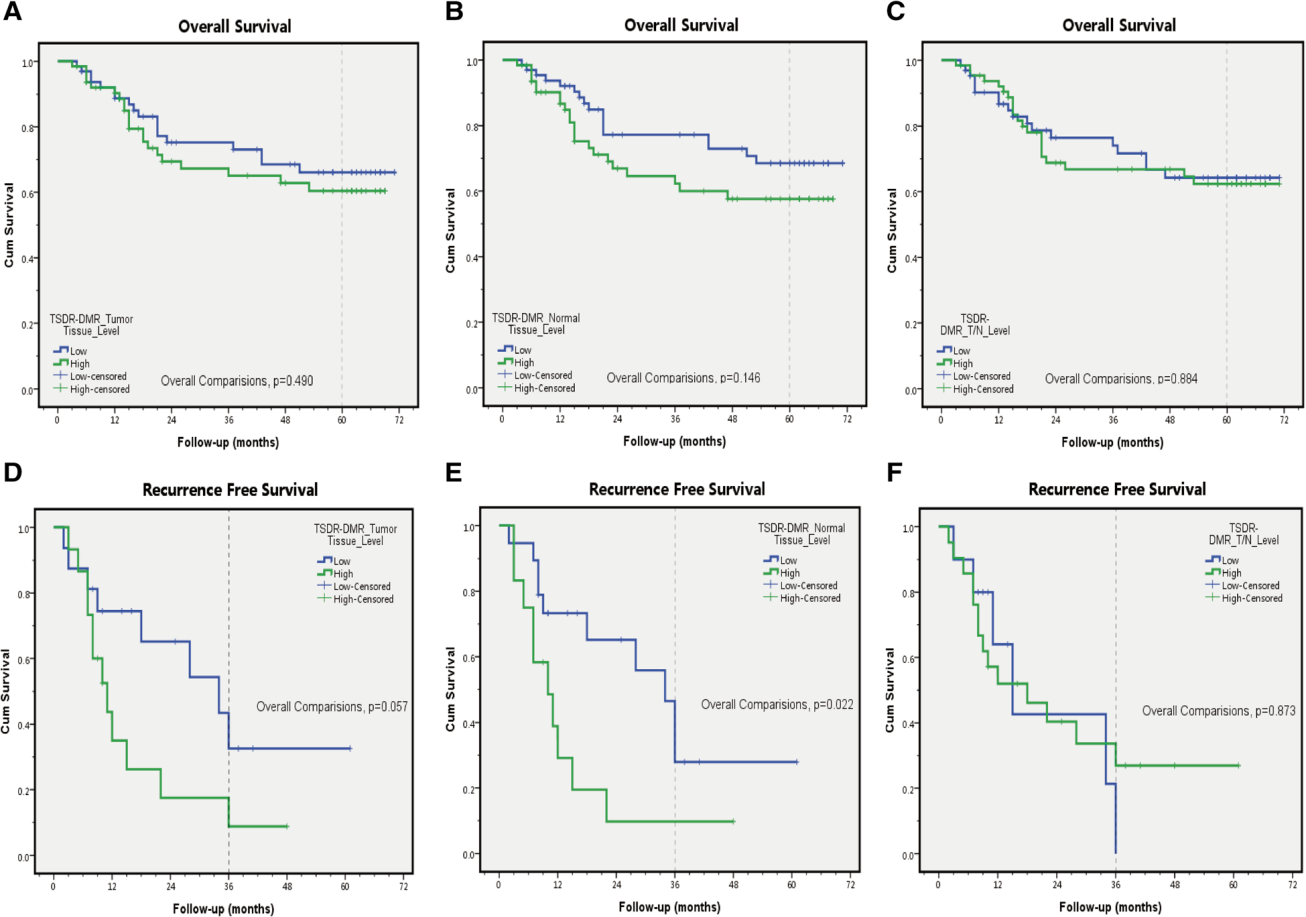
**Clinical survival analysis according to the levels of TSDR-DMR in different tissue samples.** Essentially, patients with lower DMRs, both in tumor samples (Figure 5**A**, 5**D**) and normal samples (Figure 5**B**, 5**E**), tended to have better survival when compared to patients with higher DMRs. However, the difference was significant only for RFS according to the variable DMR_N_ (Figure 5**E**, *p* = 0.022). And no significant difference was found either for OS (Figure 5**C**) or for RFS (Figure 5**F**) according to the variables of relative TSDR demethylation level (DMR_T_/DMR_N_). DMRs were classified as high or low in relation to the median values for each (see Additional file 1: Tables S1 and Additional file 2: Table S2). Log-rank (Mantel-Cox) tests were performed for the overall comparisons. Reference lines within the graphs indicate the 5th year and 3rd year for OS and RFS, respectively.

We next analyzed the clinical survival outcomes, including overall survival (OS) and recurrence-free survival (RFS), according to DMR_T_ and DMR_N_ as well as DMR_T_/DMR_N_. Overall, the patients completed a median follow-up of 54.5 (3-71) months. When compared to patients with higher TSDR-DMRs (either DMR_T_ or DMR_N_), patients with lower demethylation rates had better OS and RFS as well as a longer median survival time (Figure 5; also see Additional file 1: Tables S1 and Additional file 2: Table S2). Overall comparisons performed with log-rank tests revealed that the difference was significant only for RFS according to the variable DMR_N_ (low vs. high, *p* = 0.022, Figure 5E). This result implied that a high proportion (density) of nTregs infiltrating adjacent normal tissues other than malignant nests was associated with worse survival outcomes in patients with non-stage IV colon cancer.

### Cox correlation analysis revealed that *FOXP3*-TSDR DMR levels were not independent prognostic factor

To investigate the prognostic variables that can predict the overall or recurrence-free survival of patients with colon cancer, Cox correlation analyses were performed. Univariate analysis revealed that Differentiation (*p* = 0.038), American Joint Committee on Cancer (AJCC) stage (*p* = 0.002), Distant metastases (*p* = 0.000), and Extranodal tumor deposits (*p* = 0.000) had a significant influence on OS, while carcinoembryonic antigen (CEA) (*p* = 0.015) and DMR_N_ level (*p* = 0.030) had significant prognostic value for RFS. Further multivariate analysis, however, revealed that Distant metastasis (M_0_ vs. M_1_, hazard ratio (HR) =7.431, 95% confidential intervals (CI): 3.864-14.291, *p* = 0.000) and CEA (normal vs. elevated, HR = 4.144, 95% CI: 1.311-13.099, *p* = 0.015) were the only independent prognostic variables for OS and RFS, respectively (Table 2). This result implied that tissue-resident nTregs especially in normal tissue adjacent to tumor nests may have a negative role on anti-tumor effects; however, these influences might be limited and become complicated when their overall impacts on patient survival are studied.

**Table 2:** Cox analysis of the prognostic variables on the overall and recurrence-free survival in patients

**Prognostic variable**	**Overall survival**		**Recurrence-free survival**	
	***P *** **value**	**HR (95% CI)**	***P *** **value**	**HR (95% CI)**
Univariate analysis				
Gender, Male/Female	0.342	1.357 (0.723-2.550)	0.301	0.602 (0.230-1.576)
Age, ≤60/>60 years	0.494	0.802 (.425-1.510)	0.607	0.787 (.315-1.963)
Maximum Size, ≤5/>5 cm	0.321	1.394 (0.724-2.683)	0.700	1.207 (0.463-3.149)
Location, Left side/Right side	0.129	1.649 (0.864-3.145)	0.365	1.557 (0.597-4.062)
Differentiation, G1-G2/G3-G4	0.038*	1.959 (1.039-3.694)	0.807	0.891 (0.354-2.245)
Mucinous or signet-ring carcinoma, No/Yes	0.684	0.851 (0.391-1.851)	0.354	0.557 (0.162-1.918)
CEA, Normal/Elevated	0.054	1.874 (0.990-3.547)	0.015*	4.144 (1.311-13.099)
Tumor (T) stage, T1-2/T3-4	0.061	0.256 (0.062-1.064)	0.920	0.927 (.210- 4.085)
Nodal (N) status, N0/N1-2	0.504	0.807 (0.430-1.514)	0.121	2.140 (.818- 5.598)
Distant metastases (M), M0/M1^a^	0.000*	8.412 (4.332-16.338)		
AJCC Stage, I-II/ III-IV	0.002*	3.155 (1.533-6.490)	0.551	0.762 (.312-1.860)
Lymphatic/vascular invasion, No/Yes	0.268	1.440 (0.755-2.746)	0.455	0.691 (0.263-1.821)
Perineural invasion, No/Yes	0.354	1.444 (0.664-3.142)	0.085	0.168 (0.022-1.275)
Extranodal tumor deposit, No/Yes	0.000*	4.341 (2.216-8.504)	0.236	2.126 (0.611-7.399)
DMR_T_ level, Low/High	0.493	1.246 (0.664-2.339)	0.070	2.320 (0.934-5.761)
DMR_N_ level, Low/High	0.153	1.588 (0.843-2.994)	0.030*	2.709 (1.102-6.662)
DMR_T_/DMR_N_, Low/High	0.884	1.048 (0.558-1.967)	0.876	0.926 (0.353-2.431)
Multivariate analysis				
Distant metastases (M), M0/M1	0.000*	7.431 (3.864-14.291)		
Differentiation, G1-G2/G3-G4	0.518	1.250 (0.635-2.459)		
AJCC Stage, I-II/III-IV	0.885	1.074 (0.406-2.846)		
Extranodal tumor deposit, No/Yes	0.330	1.479 (0.673-3.248)		
CEA, Normal/Elevated			0.015*	4.144 (1.311-13.099)
DMR_N_ level, Low/High			0.286	1.733 (0.631-4.755)

*A two-tailed *p* value ≤0.05 was considered statistically significant.

^a^For Cox correlation analysis, non-stage IV colon cancer were included for RFS study, so this variable was not selected for analysis.

*Abbreviations*: HR, hazard ratio; CI, confidential intervals; CEA, carcinoembryonic antigen; AJCC, American Joint Committee on Cancer (AJCC); DMR_T_, demethylation rate in tumor tissues; DMR_N_, demethylation rate in normal tissues.

## Discussion

Epigenetic DNA methylation-based diagnostics represent a new research tool, offering various advantages over traditional methods [35]. In our study, the *FOXP3*-TSDR demethylation status could be accurately monitored and the exact proportions of nTregs in tissue samples could be estimated with high sensitivity using this MS-qPCR system. In addition, when compared to IHC, FCM, and TMA, it is far less complicated and expensive, and it can be readily applied to large numbers of archival or frozen specimens, including blood, tissue, and stool samples [35].

Using the *FOXP3*-TSDR demethylation assay, Wieczorek *et al.*[4] measured the nTreg proportions (DMR) in the peripheral blood of patients with CRC tumors (n = 27) and in that of healthy donors (n = 20); however, no significant difference was found (2.3% vs. 1.4%, *p* = 0.068). In formalin-fixed, paraffin-embedded (FFPE) tissue samples, significantly higher DMR was noted in tumor tissues of patients with CRC (n = 15) than in the adjacent normal colonic mucosa (n = 10) (6.3% vs. 1.5%, *p* < 0.001). This was a pilot study aimed at investigating the proportion of nTregs in different samples by using this epigenetic marker; however, no valuable prognostic conclusions could be made due to the statistical power. With a larger sample size, our data reinforced the feasibility of nTreg identification utilizing this *de novo* strategy in CRC-related research.

When compared to nTregs, an extremely low DMR was detected among six CRC cell lines in the present study (Figure 2A). By using MS-qPCR, Lucas and colleagues analyzed the FOXP3i1 (TSDR) demethylation status in various cells lines of malignant carcinomas, including CRC, Non-small cell lung cancer (NSCLC), and melanoma. They found that none of the seven CRC cell lines and most of the other lines mentioned above contained a substantial level of demethylated *FOXP3*-TSDR [19]. Baron and colleagues also confirmed this differential demethylation status of TSDR between nTregs and other blood cell subtypes as well as various non-hematopoietic tissues [14]. CRC cell lines did show detectable *FOXP3* mRNA signals, although their levels were extremely low when compared to nTregs (Figure 2B). We could not exclude the possibility of that FOXP3 might also function as a potential transcriptional suppressor of oncogene in CRC cells as in breast cancer cells [36-38] and prostate cancer [39,40]. Actually, it has been reported that FOXP3 expression appears widespread in normal epithelia [41] and malignant cells, such as glioblastoma [42], ovarian cancer [43], NSCLC [44], or advanced gastric cancer [45]. It was also found both in CRC cells *in vivo*[46] and in colon cancer cell line CaCo2 [47], HCA 2.6 and HCA 3.2 [48]*in vitro*. Obviously, however, the concerns fell out the scope of this study, in which our focus was on the expression levels of *FOXP3*-TSDR and regarding it as a surrogate marker for identification pericancerous infiltrating nTregs.

The range of DMR calculated in the present study using the SYBR Green method was similar to that described in reports by Wieczorek *et al. *[4], in which sequence-specific probes were adopted in an MS-qPCR system. Additionally, the relative demethylation levels in the present study (DMR_T_/DMR_N_, median: 2.35) were comparable to that reported by Salama *et al.* (FOXP3^+^ T_T_/FOXP3^+^ T_N_, median: 2.64), which was evaluated by IHC in TMA samples [30]. This consistence indicated a similar ratio of the nTregs (demethylated TSDR status) with Tregs (FOXP3+) in tumor tissues *versus* normal tissues. However, one of the shortage in this study was the unavailability of the DMR data of colonic mucosa from the normal controls, like the others [4]. Further work might be required to judge the differential TSDR-DMR expression among tissues from healthy volunteers, tumor and the corresponding normal tissues from patients with CRC.

Tregs found at tumor sites contain thymus-derived nTregs and iTregs converted from CD4^+^CD25^-^ T cells. Their accumulation may be due to the proliferation of pre-existing Tregs in the tumor microenvironment, the recruitment of Tregs from periphery, and the *de novo* conversion of tumor-infiltrating CD4+ lymphocytes into iTreg [25,49,50]. Our results (Figure 3) were in agreement with previous reports that significantly more FOXP3+ Tregs were found in tumor tissues than that in corresponding adjacent normal mucosa [27,28,30]; and our data reinforced the fact that a majority of these suppressive Tregs are functional nTregs [51].

Our data indicated that a higher level of tissue-resident nTregs infiltration, especially into normal tissues adjacent to tumors (higher DMR_N_), was correlated with worse clinicopathological features (distant metastases) (Figure 4). Higher DMRs also tended to be associated with a shortened survival time, although only RFS differences in DMR_N_ were found to be significant after overall comparisons (Figure 5E). Our findings regarding the influence of nTregs on patients with CRC are partially in agreement with previously findings [27-30]; But others also reported different conclusions [31-34]. Although the reasons for these discordant results remain unclear, the adaptive immune response is thought to play an important and complicated role in promoting or suppressing the progression of CRC [30].

Salama *et al.*[30] were the first to report the prognostic significance FOXP3+ Tregs in normal colonic mucosa from patients with CRC. One of their remarkable findings was the opposite prognostic significance of high densities of FOXP3+ Tregs in tumor tissues (better survival) and in adjacent normal mucosa (worse survival). Partially in agreement with their findings, our data also indicated that higher levels of nTregs in normal colonic mucosa (Higher DMR_N_), but not in tumor nests, were significantly correlated with worse clinical features. FOXP3+ nTregs in normal tissues might have a negative effect on the anti-tumor response, thus explaining their association with worse prognosis [30]. However, this association was found to be significantly opposite after tissue normalization (DMR_T_/DMR_N_) (Table. 1). This statistical result might be due to the relatively less difference-value of mean ranks in DMR_T_ than that in DMR_N_, and no meaningful clinical interpretation could be made under this situation.

Correale P *et al.*[31] also reported that patients with reduced intraepithelial CD3+ T-cell densities in the tumor had reduced disease-free survival times (DFS) (HR: 1.87; 95% CI: 1.10, 3.16; *p* = 0.018). However, the intraepithelial FOXP3+ cell density in tumor nests was not prognostic. In the present study, univariate survival analysis confirmed that certain conventional histopathological markers were related with poor survival outcomes, including OS and RFS (Figure 5, Table 2). Only DMR_N_ was found to be a prognostic variable for RFS in patients with non-stage IV colon cancer by univariate analysis; however, it failed to be an independent factor after multivariate analysis. This result may be partially attributed to sampling errors, and further investigation might be required.

Nishikawa *et al.* concluded that, in CRC, the obvious prognostic contradiction associated with FOXP3+ Treg infiltration might be attributed to the different compositions of FOXP3+ T-cell subpopulations in altered tumor types and tissue sites [52]. FOXP3+ T cells infiltrating into colon cancers contain higher frequencies of effector nTreg cells (CD45RA^-^FOXP3^hi^CD25^hi^) as well as non-Treg cells (CD45RA^-^FOXP3^lo^CD25^lo^)^20^. The latter ones are capable of secreting pro-inflammatory cytokines [15,53,54], which could contribute to the improved prognosis of some patients with colon cancer even when high densities of total FOXP3+ T cells are present. Terzic *et al.* proposed the hypothesis of a septic microenvironment in colon cancer. By suppressing the inflammation and immune responses resulting from bacterial invasion, FOXP3 + Tregs could in fact be anti-tumorigenic [55]. Further functional studies of Tregs in tumor and adjacent normal tissues may be required to discover their exact role in the antitumor response.

Regarding gender, our data were consistent with the previous study. Sinicrope *et al.*[28] also detected higher levels of intratumor FOXP3 expression in female patients with CRC. Wieczorek *et al.*[4] reported similar results of slightly higher TSDR-DMR in female healthy controls versus male healthy controls. In female patients or healthy controls, one of the two *FOXP3*-TSDR alleles is methylated as a result of X-inactivation [5,56]. This might partially explain the gender difference or bias in TSDR-DMR when corrected with a factor of 2. However, there is no exact explanation for these findings, and further investigation is required.

## Conclusions

In conclusion, the *FOXP3*-TSDR demethylation status could differentiate nTregs from non-nTregs, suggesting that this epigenetic status might be a promising surrogate biomarker for the identification of nTregs in clinical research when using archival CRC samples. A significantly higher TSDR-DMR and *FOXP3* mRNA as well as protein expression level were found in tumor sites versus normal ones, implying that abnormal recruitment of nTregs occurred at tumor sites. A higher *FOXP3*-TSDR DMR in adjacent normal tissues, but not in malignant tissues, was found in patients with distant metastases; this was also associated with worse recurrence-free survival. Further analysis indicated that nTregs might have a negative role in anti-tumor effects, although their impacts on the survival outcomes may be limited and complicated.

## Methods

### Patient population, tissue samples, and clinicopathological variables

A total of 130 colon carcinoma samples were obtained after approval was granted by the Medical Ethics Committee of Fudan University Shanghai Cancer Center. Samples were retrieved from consecutive, surgically treated patients (78 males and 52 females with stage I-IV colon cancer) between January and December 2008. In this study, only patients who underwent colectomy for colon cancer without chemotherapy or radiotherapy before surgery were selected, no matter their TNM stage. Differentiation grading and TNM classification for colon cancer were confirmed according to the criteria described in the AJCC Cancer Staging Manual (7th edition, 2010). Fresh colon tumor tissues coupled with corresponding normal colonic tissues were obtained immediately after surgery, washed twice with chilled phosphate-buffered saline (PBS), immediately stored in liquid nitrogen, and kept at -80°C in our tissue bank for further use.

The patients’ electronic medical records were reviewed. Various clinicopathological variables were investigated. Clinical survival outcomes, including OS and RFS, were also studied. In this study, OS was calculated from the time when the patient was diagnosed until their death from any cause; for a non-stage IV patient with R_0_ resection, RFS was computed from the time when the patient was diagnosed to the first evidence of recurrence or metastasis. The last follow-up date was set as Dec 31, 2013.

### Isolation of PBMCs, purification of nTregs, and differentiation of iTregs

Human primary cell isolation and culture were performed as described previously [57,58]. Briefly, PBMCs were isolated by Ficoll-Hypaque (Seromed, Biochrom KG, Berlin, Germany) from buffy coats of healthy male blood donors at the Shanghai Blood Center. nTregs were separated with a FACSAria II cell sorter (BD Biosciences, USA) using the monoclonal antibodies anti-CD4-FITC, anti-CD25-PE, and anti-CD127- PE-Cy7. Naïve T cells were gated from a population of CD4+ CD25- effector T cells and separated with anti-CD45RA-Percp-CY5.5 (all from BD Biosciences, USA). Induced Tregs (iTregs) were induced from naïve T cells with recombinant TGF-β (5 ng/ml) and IL-2 (100 U/ml). The purified human nTregs and iTregs were expanded with anti-CD3/CD28 beads (Invitrogen, USA) and 500 U/ml IL-2 (R&D, USA). Both cell types were cultured with X-VIVO 15 medium (Lonza, Cologne, Germany) supplemented with 10% heat-inactivated human AB serum (Irvine Scientific, USA), 1% GlutaMax, and 1% NaPyr (Both from GIBCO, Life Technologies, USA). Cell fraction purity was determined using intracellular FOXP3 staining with FOXP3-PE-A (eBioscience, USA), following the manufacturer’s instructions. FACS data were then analyzed using FlowJo software (Tree Star, USA).

### Other cell Lines and cell culture conditions

HEK293T cells and six human colorectal cancer cell lines (RKO, Colo-320, LS-174T, SW480, SW620, and HCT 116) were utilized. All lines were obtained from the Type Culture Collection of the Chinese Academy of Sciences (Shanghai, China) within 6 months, where they were characterized by cell vitality detection, DNA fingerprinting, mycoplasma detection, and isozyme detection. The RKO, LS-174T, and HCT 116 cell lines were cultured in DMEM medium; the Colo-320 line was cultured in RPMI-1640 medium; and the SW480 and SW620 lines were cultured in L-15 medium. All media contained 10% FBS, and all media and FBS were purchased from GIBCO® (Life Technologies, USA).

### Genomic DNA isolation, bisulfite conversion, and MS-qPCR

Genomic DNA (gDNA) was isolated using DNA isolation kits (ZYMO Research, USA). For cell and tissue samples, the protocols for cultured cells or solid tissues were followed, respectively. Bisulfite treatment of 0.5-1 μg genomic DNA was performed using methylation kits (ZYMO Research, USA) according to the manufacturer’s instructions. MS-qPCR was performed using SYBR Green reagent (Thermo Scientific, USA). Primers for methylation-specific and demethylation-specific *FOXP3* were designed using MethPrimer [59]. Real-time PCR was performed in a final reaction volume of 10 μL using the ABI Prism 7900T Sequence Detection System (Applied Biosystems, USA), containing 25 pmol each of methylation or demethylation-specific forward and reverse primers for *FOXP3*-TSDR and 25-50 ng of bisulfite-treated genomic DNA template. Cycling conditions and primers for TSDR are listed (see Supplementary Information, Additional file 3: Table S3).

The demethylation rate (DMR) of *FOXP3*-TSDR was computed using a formula described previously [60,61]: 100/[1 + 2^(CtTG- CtCG)^] × 100%, where Ct_TG_ represents the cycle threshold achieved with TG (demethylated) primers and Ct_CG_ represents the cycle threshold achieved with CG (methylated) primers. For female patients, this rate was corrected by a factor of 2 because one of the two TSDR alleles is methylated as a result of X inactivation [5,56,60,61].

### RNA isolation, reverse transcription, and real-time qPCR

Total RNA was isolated from cultured cell lines or tissue samples using the TRIzol® Reagent (Life Technologies, USA) according to the manufacturer's instructions. RNA was quantified, and complementary DNA (cDNA) was reverse transcribed with the RT reagent kit (Takara, Japan) according to the manufacturer's protocol. RT-PCR was performed using the SYBR Green reagent (Thermo Scientific, USA). For quantitative PCR, 5 ng of the RT reaction was used in a 10-μL reaction volume, and amplification was performed using the ABI Prism 7900T Sequence Detection System. The cycling conditions and primers for amplification of β-actin and FOXP3 are listed (see Additional file 3: Table S3). RT-PCR products were verified by DNA sequencing. DL500 (Takara Bio Inc., Japan) was used as DNA marker for agarose gel electrophoresis.

### Tissue homogenization, lysis, and Western blotting

Tissue samples (approximately 130-150 mg) were lysed at 4°C using a homogenizer (Precellys™ 24, Bertin, France) in 2-mL tubes prefilled with magnetic beads and RIPA lysis buffer (1 mM Na_3_VO_4_, NaF, PMSF and 1% protease inhibitor cocktail [Thermo Scientific, USA]). After measurement of the protein concentration using the BCA Protein Assay Kit (Thermo Scientific, USA), protein samples (60-80 μg) were loaded and separated on 10% acrylamide gels for SDS-PAGE. Subsequently, the proteins were transferred to 0.25-μm PVDF membranes. The membranes were then incubated with a mouse monoclonal antibody against human FOXP3 (ab22510, Abcam, USA) at a concentration of 4 μg/mL (1:250) overnight at 4°C. An anti-GAPDH mouse antibody was simultaneously used as a loading control (1:2000 dilution; Suji-tech, Beijing, China). Then, the membrane was incubated with an HRP-conjugated secondary antibody (1:5000 dilution, Suji-tech, Beijing, China) for 1 hour at room temperature. All blots were visualized using an ECL Western Blotting Substrate (Thermo Scientific, USA) and were quantatively analyzed by ImageJ 1.46 (National Institute of Health, USA) [62].

### Statistical analysis

Normality tests were performed to tests whether the data were normally distributed. Median values of the DMRs were adopted as cut-off points to define low or high levels of *FOXP3-*TSDR demethylation. Prognostic factors were determined using Cox regression analysis. Kaplan–Meier curves were used to assess the influence of the TSDR demethylation status on OS and RFS. The differences were tested using the log-rank test. Statistical analyses were performed using SPSS v.20.0 (IBM Corp., USA). A two-tailed *p*-value lower than 0.05 was considered statistically significant.

## Consent

Written informed consents were obtained from the patients for the publication of this report and any accompanying images.

## Abbreviations

Tregs: Regulatory T cells; CRC: Colorectal cancer; FOXP3: Transcription factor forkhead box P3; TSDR: Treg-specific demethylated region; DMR: Demethylation rates; HR: Hazard ratio; CI: Confidential intervals; AJCC: American Joint Committee on Cancer; DMR_T_: Demethylation rate in tumor tissues; DMR_N_: Demethylation rate in normal tissues; CEA: Carcinoembryonic antigen; OS: Overall survival; RFS: Recurrence-free survival.

## Competing interests

The authors have no conflict of interest to disclose.

## Authors’ contributions

CHZ, BL, SJC, ZYL, and YX conceived and designed the study. CHZ, YWW, QGL and HTZ carried out the most experiments, ZYL and BL participated in the FACS and FCM. CHZ, QGL, JJP and PW analyzed and interpreted the survival data. CHZ drafted the manuscript. SJC, BL and YX revised the manuscript critically for important intellectual content. All authors read and approved the final manuscript.

## Authors’ information

Sanjun Cai, M.D., Professor of Surgical Oncology. Chair of Committee of Colorectal Cancer, the Chinese Anti-cancer Association. Chief Scientist of Colorectal Cancer Center, Fudan University. Chief of Department of Colorectal Surgery at Fudan University Shanghai Cancer Center. Email: caisanjun@gmail.com.

Bin Li, Ph.D., Professor of Immunology. Senior Scientist and Principal Investigator of Unit of Molecular Immunology at Key Laboratory of Molecular Virology & Immunology, Institut Pasteur of Shanghai, Shanghai Institutes for Biological Sciences, Chinese Academy of Sciences. Email: binli@sibs.ac.cn.

Changhua Zhuo, M.D., Attending Surgeon of Surgical Oncology at Fujian Provincial Cancer Hospital. Ph.D. Candidate of Oncology, Shanghai Medical College, Fudan University. Email: czhuo12@fudan.edu.cn.

## Supplementary Material

Additional file 1: Table S1 Survival studies for stage I-IV patients according to different variables in overall survival. Click here for file

Additional file 2: Table S2 Survival studies for non-stage IV patients according to different variables in recurrence-free survival. Click here for file

Additional file 3: Table S3 Primers and cycling conditions for real-time quantitative PCR assays. Click here for file
