# Supplementary material for: Higher FOXP3-TSDR demethylation rates in adjacent normal tissues in patients with colon cancer were associated with worse survival
Source: Mol Cancer. 2014 Jun 18;13:153. doi: 10.1186/1476-4598-13-153 (PMC4074420; doi:10.1186/1476-4598-13-153)
Supplement: Additional file 1: Table S1 — Survival studies for stage I-IV patients according to different variables in overall survival. [file 1476-4598-13-153-S1.doc]

**Table S1.** Survival studies for stage I-IV patients according to different variables in overall survival

| **Variables** | **Cut-off ^a^** | **Level** | **2-year survival rate** | **3-year survival rate** | **5-year survival rate** | **Estimated mean ^b^ (SE) (months)** | **95%** confidential intervals | | **Overall comparisons (log rank)** | |
| --- | --- | --- | --- | --- | --- | --- | --- | --- | --- | --- |
| DMR_T_ | 2.730% | Low | 0.772 | 0.661 | 0.500 | 54.4 (3.3) | 47.9-60.9 | *x^2^*=0.477, *p*=0.490 | |  |
|  |  | High | 0.755 | 0.628 | 0.500 | 49.5 (3.5) | 42.8-56.3 |  | |  |
| DMR_N_ | 1.015% | Low | 0.772 | 0.730 | 0.564 | 56.2 (3.1) | 50.0-62.3 | *x^2^*=2.111, *p*=0.146 | |  |
|  |  | High | 0.646 | 0.623 | 0.564 | 47.5 (3.6) | 40.4-54.6 |  | |  |
| DMR_T_/DMR_N_ | 2.350 | Low | 0.740 | 0.740 | 0.486 | 53.5 (3.4) | 46.7-60.3 | *x^2^*=0.021, *p*=0.884 | |  |
|  |  | High | 0.668 | 0.646 | 0.512 | 51.8 (3.4) | 45.1-58.6 |  | |  |

^a^ The median values for these variables were adopted as cut-off points to differentiate low or high levels of demethylated FOXP3-TSDR.

^b^ The median survival time could not be estimated as all of these three survival rates were higher than 0.5; therefore, the mean values were adopted for OS analysis.

A two-tailed *p* value ≤0.05 was considered statistically significant.
