# Supplementary material for: Higher FOXP3-TSDR demethylation rates in adjacent normal tissues in patients with colon cancer were associated with worse survival
Source: Mol Cancer. 2014 Jun 18;13:153. doi: 10.1186/1476-4598-13-153 (PMC4074420; doi:10.1186/1476-4598-13-153)
Supplement: Additional file 2: Table S2 — Survival studies for non-stage IV patients according to different variables in recurrence-free survival. [file 1476-4598-13-153-S2.doc]

**Table S2. Survival studies for non-stage IV patients according to different variables in recurrence-free survival**

| **Variables** | **Cut-off ^a^** | **Level** | **1-year survival rate** | **2-year survival rate** | **3-year survival rate** | **Estimated median (SE) (months)** | **95%** confidential intervals | | **Overall comparisons (log rank)** | |
| --- | --- | --- | --- | --- | --- | --- | --- | --- | --- | --- |
| DMR_T_ | 2.730% | Low | 0.652 | 0.543 | 0.326 | 34.0 (11.2) | 12.0-56.0 | x^2^=3.611, *p*=0.057 | |  |
|  |  | High | 0.350 | 0.083 | 0.083 | 11.0 (2.5) | 6.1-15.9 |  | |  |
| DMR_N_ | 1.015% | Low | 0.652 | 0.559 | 0.279 | 34.0 (4.0) | 26.1-41.9 | x^2^=5.262, *p*=0.022* | |  |
|  |  | High | 0.292 | 0.097 | 0.097 | 10.0 (3.0) | 4.0-16.0 |  | |  |
| DMR_T_/DMR_N_ | 2.350 | Low | 0.427 | 0.213 | 0.000 | 15.0 (3.9) | 7.2-22.7 | x^2^=0.025, *p*=0.873 | |  |
|  |  | High | 0.462 | 0.337 | 0.269 | 18.0 (8.0) | 2.3-33.7 |  | |  |

^a^ The median values for these variables were adopted as cut-off points to differentiate low or high levels of demethylated FOXP3-TSDR.

*A two-tailed *p* value ≤0.05 was considered statistically significant.
