# Supplementary material for: Higher FOXP3-TSDR demethylation rates in adjacent normal tissues in patients with colon cancer were associated with worse survival
Source: Mol Cancer. 2014 Jun 18;13:153. doi: 10.1186/1476-4598-13-153 (PMC4074420; doi:10.1186/1476-4598-13-153)
Supplement: Additional file 3: Table S3 — Primers and cycling conditions for real-time quantitative PCR assays. [file 1476-4598-13-153-S3.doc]

**Table S3.** Primers and cycling conditions for real-time quantitative PCR assays

| **Array** | **Primer sets** | **Primer sequence** | **Product size** | **Cycle condition** |
| --- | --- | --- | --- | --- |
| MS-qPCR | *FOXP3-*TSDR demethylation-specific | Forward: 5'- TAGGGTAGTTAGTTTTTGGAATGA-3'  Reverse: 5'- CCATTAACATCATAACAACCAAA-3' | 118 bp | Preheating: 98 °C for 10 min;  40 cycles of 98 °C for 15 seconds  followed by 1 min at 60 °C. |
|  | *FOXP3-*TSDR methylation-specific | Forward: 5'-CGATAGGGTAGTTAGTTTTCGGAAC-3'  Reverse: 5'- CATTAACGTCATAACGACCGAA-3' | 113 bp |  |
| RT-PCR | FOXP3 | Forward: 5'-TCCCAGAGTTCCTCCACAAC-3’  Reverse: 5’-ATTGAGTGTCCGCTGCTTCT-3’ | 122 bp | Preheating: 98 °C for 10 min;  40 cycles of 98 °C for 15 seconds  followed by 1 min at 60 °C. |
|  | β-actin | Forward: 5’-GGACTTCGAGCAAGAGATGG-3’  Reverse: 5’-AGCACTGTGTTGGCGTACAG-3’ | 234 bp |  |
